# Search and Selection of Probiotics That Improve Mucositis Symptoms in Oncologic Patients. A Systematic Review

**DOI:** 10.3390/nu11102322

**Published:** 2019-10-01

**Authors:** José Antonio Picó-Monllor, José Manuel Mingot-Ascencao

**Affiliations:** 1 Universidad Miguel Hernández, 03202 Elche, Spain; 2 Korott, s.l., 03801 Alcoy, Spain

**Keywords:** mucositis, probiotics, neoplasms

## Abstract

Mucositis is a common and severe adverse effect of radiotherapy and/or chemotherapy treatments applied to oncologic patients. The development of effective therapies and adjuvant treatments to increase their efficacy and reduce adverse effect is a priority in cancer therapy. Probiotics are non-pathogenic live microorganisms that when ingested in adequate amounts can colonize the intestinal tract promoting the restoration of a healthy gut microbiota and contributing to all its functions including the maintenance of the integrity of the mucosa and the modulation of the immune system. In order to check the possible efficacy and safety of these microorganisms to prevent or ameliorate mucositis′ symptoms, we have systematically searched the bibliographic databases MEDLINE (via Pubmed), EMBASE, The Cochrane library, Scopus, Web of science, and Latin American and Caribbean Literature in Health of Sciences (LILACS) using the descriptors “Mucositis”, “Probiotics”, “Neoplasms”, “Humans”, and “Clinical Trials”. After applying our inclusion and exclusion criteria, 15 studies were accepted for review and critical analysis. Our analysis suggests that a combination of *Bifidobacterium longum*, *Lactobacillus acidophilus*, *Bifidobacterium breve*, *Bifidobacterium infantis,* and *Saccharomyces boulardii* could be a good combination of probiotics to reduce incident rates of mucositis or ameliorate its symptoms in chemo or radiotherapy treated patients.

## 1. Introduction

Mucositis is a common and severe adverse effect of chemotherapy or radiotherapy treatments applied to oncologic patients. It can affect any part of the mucosa layer of the gastrointestinal tract from the mouth to the anus. It happens in 20%–40% of the patients undergoing conventional chemotherapy, 80% of patients receiving high chemotherapy doses, 60%–80% of patients receiving hematopoietic cell transplants, and in almost all patients with squamous cell carcinoma of the head and neck undergoing radiotherapy [1,2,3,4].

### 1.1. Pathogenesis

Although mucositis is a process that occurs continuously over time, it is frequently divided into five stages, namely, initiation, primary damage response, signal amplification, ulceration, and healing [5]. The initiation or first damage induced by chemo or radiotherapy involves DNA damage and the generation of reactive oxygen species in cells of both, the basal epithelium and the submucosa layer. These events lead to a primary damage response in all the mucosa layers, by the activation of several transcription factors, such as nuclear factor kappa-light-chain-enhancer of activated B cells (NF-κB) [6], that regulate the expression of many genes involved in inflammation and mucosa damage such as interleukin 1-beta (IL-1β), tumor necrosis factor alfa (TNF-α), interleukin 6 (IL-6), and the metalloproteases (MMP) MMP1 and MMP3 whose activity degrades subepithelial matrix and the epithelial basement membrane [7]. Positive-feedback loops between some of these molecules, as the activation of NF-κB by TNF-α or MMP1 and MMP3 by TNF-α and IL-1β, further amplify the activated signalling pathways finally leading to the mucosa damage or ulcer. Loss of mucosa integrity causes severe pain and increases the risk of bacteria translocation and, therefore, the risk of bacteremia and sepsis. Finally, once the therapy finishes, in most of the cases the ulcer heals.

### 1.2. Assessment Scales Mucositis

Clinically, mucosa injury can be assessed by different criteria. One of the most widely used for mucositis evaluation in clinical trials has been stablished by the World Health Organization (WHO) [8]. By these criteria, mucositis is classified, as severity increases, from grade 0 to grade 4. Mucositis is classified as grade 0 when the patient shows no signs or symptoms; grade 1 when the patient has painless ulcers, edema, or mild pain; grade 2 when there is painful erythema, edema, or ulcers but the patient is able to eat; grade 3 when the patient has the same symptoms as in grade 2 but is unable to eat; and, finally, grade 4 when parenteral or enteral feeding is needed (Table 1). Severe mucositis usually requires a decrease in the dose of treatment or even its temporary interruption which negatively affects the prognosis of patients [9,10]. Besides the negative effects of mucositis in cancer patients, this pathology also has a significant economic impact due to the costs associated with the treatment of symptoms, nutritional requirements, treatment of secondary infections, and hospitalization expenses [9,10,11].

### 1.3. Drugs

Therapy options for mucositis treatment are continuously been developed and many kinds of drugs, targeting different molecular pathways involved in the pathology or alleviating some of its symptoms, are currently been used [12]. This battery of drugs includes reactive oxygen species, protective molecules such as amifostine and n-acetyl cysteine, a keratinocyte growth factor inhibitor, palifermin, nonsteroidal anti-inflammatories such as benzydamine HCl, TNF-α inhibitors such as pentoxifylline, and mono or polyclonal antibodies against TNF-α or Il-6. However, these drugs are not very efficient [1] and the development of effective therapies or adjuvant treatments to increase their efficacy and reduce adverse effect is a priority in the cancer therapy field.

When mucositis affects the intestinal mucosa, it is usually accompanied by nausea, vomiting, diarrhea, abdominal pain, bleeding, dehydration, electrolyte imbalance, immunosuppression, weakening of the intestinal barrier, and dysbiosis [1]. The combination of a damaged intestinal mucosa, dysbiosis, and immunosuppression favors the translocation and subsequent bacterial spread that can cause systemic infections that constitute a severe complication in cancer patients [13]. In order to prevent or treat these infections, patients are treated with broad-spectrum antibiotics. These antibiotics further weaken the intestinal microbiota and induce a greater degradation of the mucosa, with the latter favoring the possible bacterial translocation.

### 1.4. Microbiota

The human intestinal microbiota plays a fundamental role in the maintenance of homeostasis and intestinal integrity. It is made up of billions of microorganisms present in our digestive tract with which we maintain a mutually beneficial relationship that has developed over millions of years. This set of microorganisms is starting to be considered as another organ of our body as it is involved in many functions such as the proper metabolism of several nutrients, the maintenance of the intestinal mucosa, the modulation of inflammatory and immune responses, and the reduction of oxidative stress. In addition, it constitutes an essential barrier against pathogenic organisms being crucial to avoid colonization of the intestinal mucosa by exogenous microbes and to prevent their translocation and subsequent invasion of other organs and tissues [14]. Patients undergoing chemotherapy or radiotherapy have altered the intestinal microbiota and these alterations could be involved in the development of mucositis and the aggravation of some of its symptoms as diarrhea and bacteremia. Thus, keeping or restoring a healthy gut microbiota could be a key factor in decreasing the incidence rates of mucositis or ameliorate its symptoms.

### 1.5. Probiotics

Probiotics are non-pathogenic live microorganisms which when administered in adequate amounts confer health benefits on the host [15]. The number of studies in which they have successfully been used to attenuate or prevent symptoms related to several pathologies is constantly growing and consumers are increasingly aware of their existence and their benefits. This has made the probiotics sector one of the fastest growing sectors among functional foods with a market valued at 32.06 US billion dollars in 2013 and expected to reach 46.55 US billion dollars by 2020. Since more and more people and healthcare staff are concerned about probiotics benefits, their consumption is constantly growing [16].

The main objective of this work is to systematically review available data related with the efficacy and safety of the use of probiotics to treat or prevent mucositis in oncologic patients and identify the best putative candidates for this purpose.

## 2. Materials and Methods 

### 2.1. Source of Data Collection

The aim of this descriptive study is to critically and systematically review articles published in the following data bases: MEDLINE (via PubMed), EMBASE, SCOPUS, Cochrane Library Plus, Institute for Scientific Information (ISI)-Web of Science, and Latin American and Caribbean Literature in Health Sciences (LILACS).

### 2.2. Information Processing

Thesaurus, a tool developed by the *U.S. National Library of Medicine*, was used to determine the data search. The entry terms “Mucositis”, “Probiotics”, and “Neoplasms”, in text format, were used as descriptors in the title and abstract. The final search equation for searching MEDLINE data base, via PubMed, was developed using Boolean connectors and the filters “Humans” and “Clinical Trial” with the following results:

(“Neoplasms”[Mesh] OR “Neoplasms”[Title/Abstract] OR “Neoplasia”[Title/Abstract] OR “Neoplasias”[Title/Abstract] OR “Neoplasm”[Title/Abstract] OR “Tumors”[Title/Abstract] OR “Tumor”[Title/Abstract] OR “Malignant Neoplasms”[Title/Abstract] OR “Malignant Neoplasm”[Title/Abstract])) AND (“Probiotics”[Mesh] OR “Probiotics”[Title/Abstract] OR “Probiotic”[Title/Abstract])) AND (“Mucositis”[Mesh] OR “Mucositis”[Title/Abstract] OR “Mucositides” [Title/Abstract]). The same scheme was arranged after considering the different features of the remaining database.

The search was conducted from the first available date, until June 2019 (date of the last update). In addition, as a secondary search and in order to reduce the number of not retrieved studies, the bibliography of the articles selected in the first search were analysed, looking for studies not detected in the primary search.

### 2.3. Final Selection of Articles

The final selection of the articles was done based on the following inclusion criteria: The documents had to be clinical studies published in peer-reviewed journals, written in English, Spanish, or Portuguese and from which the full text could be retrieved. Also, there was an exclusion of all studies either not performed on humans or which did not focus the intervention (probiotics) on the effect over mucositis.

Relevant articles were independently selected by the two authors (J-P and J-M) of this review. In order to validate the choice of the articles, it was established that the value of the agreement between these two authors (Kappa index) had to be greater than 0.80 (a score that guarantees a very good strength of the agreement). Whenever this condition was met, possible disagreements between the authors would be resolved by consulting an expert in the field and a subsequent consensus between the authors [17].

The quality of the selected articles was evaluated according to the guidelines of the Consolidated Standards of Reporting Trials (CONSORT) [18] that contains 25 essential issues that must be described and evaluated in the publication of this type of studies. In each selected article, the items were given a “1” or “0” depending on whether they contained the information mentioned in CONSORT. If the evaluation of an item was not necessary, that point was not counted in the total score (Not Applicable = NA). When an item was made up of two points, these were independently evaluated and subsequently averaged in such a way that in no case the score of the item could be greater than one.

### 2.4. Data Extraction

The information extracted from the articles reviewed in this essay was controlled by double-entry tables which permitted the detection of errors and, at the same time, their correction by checking the original one again.

To establish the actuality of the articles, the Burton-Kebler half-period (the median age) and the Price index (percentage of articles with an age of less than five years) were calculated. The articles were grouped according to the variables under study, in order to systematize and facilitate the understanding of the results, coding the following data: First author and year of publication, study design, country where the study was conducted, study population, pathology, period in which the work was carried out, what type of intervention took place, and results obtained.

## 3. Results

**Figure 1:**
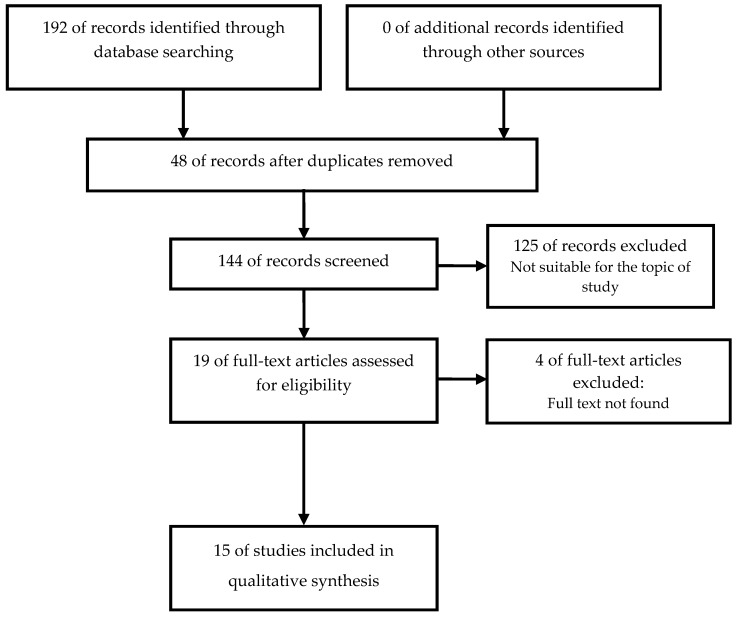
Identification and selection of studies according to Preferred Reporting Items for Systematic Reviews and Meta-Analyses (PRISMA statement [19].

When applying the described search criteria, a total of 192 references were retrieved: 88 (45.83%) in EMBASE, 42 (21.87%) in Scopus, 38 (19.79%), 18 (9.38%), in MEDLINE, 6 (3.12%) in Web of science, and 0 in LILACS. From all the identified articles, 48 (25%) were rejected because they were duplicated in more than one bibliographic database. After applying the inclusion and exclusion criteria (Figure 1), of the remaining 144 (75%), 15 studies (7.81%) were accepted for review and critical analysis (Table 2).

The agreement on the pertinence of the selected studies was 100%. The obsolescence of the chosen articles, according to the Burton-Kebler index, was 8.5 years, with a Price index of 27.7%. 

The quality assessment of the articles selected for this review by CONSORT [18] (Table 3) obtained a score between 9.5 and 24.5 over a maximum score of 25 items with a median equal to 17. The calculation of the Kappa coefficient gave a measure of agreement in the selection of articles by the two authors of 98%.

Most of the studies were randomized placebo controlled clinical trials (14; 93%) [20,21,22,23,24,26,27,28,29,30,31,32,33,34]. Two studies, a triple cohort study with published results of two cohorts [26] and a pilot study [25], stand out. The origin of the studies was varied with a slight predominance of Asian countries, such as China [20,21,22], Japan [32], and India [29], (five studies), and the rest were distributed in different countries like Italy (two studies) [23,30] and Brazil [31], Netherlands [25], Australian [24] Ireland [28], Egypt [27], Sweden [33] and Finland [34] with one study each one. A multicenter study in the USA and Republic of Korea stands out [26].

On average, the age of the population included in the studies was between 6.5 and 72 years and the number of subjects per study oscillated between 20 [24] and 173 [26]. Regarding the distribution by sex, it was observed that male sex prevailed [20,21,22,23,24,25,26,27,29,30,31,33]. In one study [28] the sex of the participants was not specified.

Most studied pathologies were colorectal cancer (CRC) [21,22,23], concurrent chemoradiotherapy (CCRT) [20] resection colon (RC) [31,33], non-small cell lung cancer (NSCLC) [26] head and neck cancer (HNC) [29,30], ulcerative colitis (UC) [27,28] affecting mucous membranes and both oral mucositis (OM) and gastrointestinal mucositis (GIM). Also, biomarkers of inflammation and cytokine levels in gastrointestinal and extra-intestinal inflammatory disorders [28] or in the context of colorectal carcinogenesis [24] were assessed in healthy volunteers. The follow-up period in the selected works ranged between five days before rectal colon surgery [21] and 39 months [30] after CCRT.

The intervention applied was very similar in all studies, as their main objective was to determine, mainly, the influence of probiotic intake on the intestinal microbiota and on different symptoms such as mucositis, diarrhea, and bacterial translocation in patients undergoing different radio and chemotherapy treatments. One study [24] used healthy patients to assess probiotic intake and characterized the luminal and biological consequences of these supplements in the context of colorectal carcinogenesis.

The probiotics used in the different interventions were, mainly, gram positive bacteria of the genus *Bifidobacterium* and *Lactobacillus*, highlighting the species *B. longum, B. infantis, B. breve,* and *B. lactis* and *L. acidophillus, L. plantarum, L. johnsonii, L. delbrueckii, L. fermentum,* and*, L. lactis*, [20,21,22,23,24,32,33,34], respectively. Other species of the *Enterococcus genus, E. faecium,* and E. *faecalis* [20,21] were part of the intervention. In one study, unlike the rest, the probiotic used was the yeast *Saccharomyces boulardii* [31]. The VSL#3 trademark, composed of four *Lactobacilli* species; L*. plantarum, L. casei, L. acidophilus, and L. delbrueckii subspecies bulgaricus*, three *Bifidobacteria* species; *B. infantis, B. longum,* and *B. breve,* and one *Streptococci species; Streptococcus salivarus subespecies thermophilus*, was also used in two studies [25,26]. In some studies, the probiotics were combined with prebiotics such as: High-amylose maize starch (HAMS) [24] and lactitol [34].

### 3.1. Probiotics and Healthy People

Among the studies selected in this review, two of them [24,34] showed that probiotic consumption by healthy people could play a protective role. In one of the studies, consumption of *B. animalis lactis* and resistant starch has modified the intestinal microbiota of healthy subjects increasing the abundance of species that, at least in mouse, are protective against intestinal tumorigenesis [24]. In the other one, administration of *Lactobacillus acidophilus* strain combined with lactitol to healthy elderly has improved intestinal microbiota health and immune parameters as seen by an increase of *Bifidobacterium* species, spermidine, PGE_2_ and IgA in faecal samples [34].

### 3.2. Probiotics and CRC

Four of the selected studies [21,22,23,31] showed the protective effect of probiotics administration in colorectal cancer patients who underwent surgery. Gao et al. [21], have shown that the colonic microbiota of these patients was less abundant and rich that the one of healthy subjects and that the administration of *Bifidobacterium longum, Lactobacillus acidophilus,* and *Enterococcus faecalis* for five days, partially prevented the observed dysbiosis, increased the abundance of beneficial bacteria, and decreased the number of some pathogenic ones including species of the genus *Fusobacterium* that have been proposed as a contributing factor for CRC development. In the study by Gianotti et al. [23], treatment with different doses of two probiotics, *Bifidobacterium longum* and *Lactobacillus johnsonii*, led, in a dose dependent way, to a decrease of pathogenic microorganisms, both in colonic mucosa and faecal samples, and an increase in CD3+, CD4+, and CD8+ T lymphocytes, suggesting a better protection of probiotic treated patients against possible infections. In their study, Liu et al. [22], showed that the administration of three probiotics, *Lactobacillus plantarum*, *Lactobacillus acidophilus,* and *Bifidobacterium longum*, for sixteen days increased the diversity and abundance of the faecal microbiota, improved the integrity of the intestinal mucosa and decreased possible postoperative clinical complications, such as diarrhea and infections. Finally, Consoli et al. [31] observed in patients undergoing colon resection that the administration of *Saccharomyces boulardii* for one week decreased the mucosa expression of proinflammatory cytokines IL-1β and IL-23A and the incidence rate of postoperative infections (38.8% in the placebo group versus 13.3% in the probiotic group). However, probiotic treatments do not always improve the conditions of these patients as shown in the study by Mangel et al. [33]. The authors treated patients with a single probiotic, *Lactobacillus plantarum* 299 v for 13 days, and this treatment didn’t decrease bacterial translocation or postoperative complications. The fact that this species has been successfully used by Liu et al. [22], when combined with other probiotics suggests that either the strain 299 v is not efficient or that this species has no effect when used alone.

### 3.3. Probiotics and Mucositis

Several of the selected studies showed that probiotics can decrease the incidence rate of mucositis induced by radio and/or chemotherapy and attenuate its symptoms in patients with different kind of cancer.

In the study by Jiang et al. [20], the incidence rate and severity of oral mucositis induced by chemo and radiotherapy in patients with nasal pharyngeal carcinoma was decreased by the administration, during the whole treatment, of *Bifidobacterium longum*, *Lactobacillus lactis,* and *Enterococcus faecium*. The results suggest that the probiotics attenuated the dysbiosis and the decrease of T type lymphocytes induced by the radiotherapy. Wada et al. [32], has shown in hematological malignancies patients treated with chemotherapy, that the administration of *Bifidobacterium breve* strain Yakult tended to decrease the incidence and severity of different adverse effects such as fever, diarrhea, and days of antibiotic treatment. In a clinical trial with patients with squamous cell carcinoma of the head and neck treated with radiotherapy, Sharma et al. [29] reported that the administration of *Lactobacillus brevis* CD2 reduced the incidence of grade 3 and 4 oral mucositis (52% incidence in the probiotic group versus 77% in the placebo group). However, De Sanctis et al. [30], found in a similar study (same type of patients treated with radiotherapy) that the same probiotic treatment had no effect. The fact that De Sanctis et al. [30], took a smaller sample size than Sharma et al. [29], 75 subjects instead of 200, and that they applied a less aggressive radiotherapy, that induced a lower rate of grade 3 and 4 oral mucositis, could explain the lack of effect observed by these authors.

### 3.4. Probiotics and Other Intestinal Pathologies

The anti-inflammatory effect of probiotics has been shown in other pathologies with a clear inflammation component including intestinal pathologies as ulcerative colitis and Crohn’s disease. As an example, Hegazy et al. [27], have shown that the administration of *Lactobacillus delbrueckii* and *Lactobacillus fermentum* to ulcerative colitis patients decreased the recruitment of leukocytes to the colonic mucosa and the expression levels of different colonic inflammatory cytokines such as IL-6, TNF-α, or NF-κB p65. In another study, Groeger et al. [28], have shown a similar effect with *Bifidobacterium infantis* in three different pathologies namely, ulcerative colitis, psoriasis, and chronic fatigue syndrome (CFS). In the three cases, but not in the same way, treatment with the probiotic decreased serum levels of several proinflammatory cytokines such as IL-6, TNF-α, or C-reactive protein (CRP).

## 4. Discussion

The study of the current events/obsolescence of the chosen topic presents a moderate validity, since, from all documents that could be recovered, 40% of the articles have been published in the last seven years; Burton Kebler’s index presented a value appropriate to the expected one, while Price’s index obtained a slightly lower than expected value in the area of health science [35].

On the other hand, according to the degree of evidence and recommendation of the U.S. Preventive Services Task Force (USPSTF) [36], the controlled and randomized clinical trials (CRCT) provide the most scientific evidence for their consistent cause-and-effect relationship. The evaluation of the quality of the studies included in this review through CONSORT was acceptable with an average of 17 out of 25.

Likewise, English is the language chosen for the publication of most articles as doing so in a different language has a negative on the impact factor and quotations [37]. In addition, the number of English-speaking journals contained in databases is currently very high [38]. Regarding the population included in the studies was heterogeneous and mostly adult with the exception of the one of Wada et al. [32] who were children.

There is high interest in improving mucositis symptomatology because it represents a health problem affecting more than two million people worldwide each year [39]. Reinforcing the possible use of probiotics in mucositis, several studies have shown its effectiveness in the prevention or treatment of two other important features of mucositis namely diarrhea and an increased intestinal permeability.

Probiotics antidiarrheal effect is very well established being probably one of their best-known effects. They are effective in the prevention and/or attenuation of practically all types of diarrheas including diarrhea associated with rotavirus infection in children, acute diarrhea in children and adults, antibiotic-associated diarrhea, travellers’ diarrhea, or AIDS-associated diarrhea [40,41,42,43,44,45]. Although less well known and established, a growing number of studies, besides the one by Liu et al. [22], is showing that probiotics can enhance the intestinal permeability barrier. This has been shown, for instance, in children with Crohn’s disease [46] and in different groups of elite athletes [47,48,49].

Probiotics can also prevent or attenuate other gastrointestinal mucositis symptoms as nausea, vomiting, and abdominal pain. This effect has been widely reported when they have been used as adjuvant in treatments against *Helicobacter pylori*, mainly based in the use of antibiotics and proton pump inhibitors, that frequently cause the aforementioned side effects [50,51,52,53,54,55].

In agreement with these results, probiotics have successfully been used to prevent mucositis symptoms in animal models. In mice, administration of *Lactobacillus casei rhamnosus* [56]. *Saccharomyces boulardii* [57], *Lactobacillus acidophilus* [58], or *Bifidobacterium bifidum* [59] has decreased the mucositis induced by 5-Fluorouracil (5-FU) and a similar effect was observed in rats by the administration of either a combination of *Bifidobacterium breve*, *Lactobacillus acidophilus*, *Lactobacillus casei*, and *Streptococcus thermophilus* [60] or *Bifidobacterium infantis* alone [61,62]. Also, in rats, the administration of the probiotic mix VSL#3 has reduced the incidence and severity of irinotecan-induced diarrhea [63].

The fact that in the studies analysed in this review at least 13 different species of bacteria and one yeast species have been successfully used, either alone or in different combinations, suggests that not only a single specific strain or one single combination of probiotics is going to work but rather many combinations could be a good treatment option. In order to select the appropriate combination of strains, important factors should be considered as the fact that not all the combinations are able to prevent the adverse effects induced by all the treatments. In this sense, Lacouture et al. [26], showed, that the administration of the probiotic combination VSL#3 to advanced non-small cell lung cancer patients was unable to prevent the mucositis and the diarrhea induced by dacomitinib, a pan-human epidermal growth factor receptor (HER) inhibitor. Another important factor to be considered is the possible interference between the probiotic and different components of the patient’s treatment. Although their results were not statistically significant, Friederich et al. [25], showed that whereas either a treatment with a symbiotic (VSL#3 and inulin) or sulindac (a non-steroidal anti-inflammatory) increased Glutathione S-transferase (GST) activity and decreased proliferation rates at the colonic mucosa, the combination of both treatments had the opposite effects. 

The most frequently used probiotic in our selected studies, is *Bifidobacterium longum*. Always combined with other probiotics, it has successfully been used in four studies, three of them in patients undergoing colorectal surgery [21,22,23] and the other one in patients with nasal pharyngeal carcinoma treated with chemo and radiotherapy [20]. Mixes containing this probiotic have had several positive effects as the restoration of a normal microbiota, immune response modulation, an enhancement of the intestinal barrier and a decrease of postoperative clinical complications as diarrhea and infections.

*Lactobacillus acidophilus* has been the second most frequently used probiotic. It has been successfully used either alone, one study [34] with healthy subjects, or combined with other two strains, two studies [21,22] with subjects undergoing colorectal surgery. When combined with other probiotics, one of them was always *Bifidobacterium longum*, suggesting that, when used together, these two species seem to be efficient in mucositis prevention.

Two species, *Lactobacillus plantarum* and *Lactobacillus brevis*, have been used two times. When *L. plantarum* was used alone in patients undergoing colon resection it had no effect in the prevention of postoperative complications [33]. Only when combined with *B. longum* and *L. acidophilus* in patients undergoing surgery, its administration was efficient restoring a normal microbiota, decreasing the intestinal permeability, and preventing postoperative complications [22]. As these effects have been observed whenever *B. longum* and *L. acidophilus* are used together, it is not clear if *L. plantarum* had any effect at all.

An identical treatment with the strain *L. brevis* CD2 has been used in two studies with squamous cell carcinoma of the head and neck patients undergoing radiotherapy. Whereas in one of the studies [29] the treatment successfully decreased the incidence rate of grade 3 and 4 mucositis, in the other one was totally ineffective [30]. Although the number of patients and the applied radiotherapy was different between both studies, the disagreement between the results shows that this probiotic is not always effective.

Ten probiotics have only been tested once. Six of them (Lactobacillus lactis, Enterococcus faecium, Enterococcus faecalis, Lactobacillus johnsonii, Lactobacillus delbrueckii, and Lactobacillus fermentum) have been used in combination with other strains making almost impossible to guess their specific contribution to the observed effects. In addition, two of these species, E. faecium and E. faecalis are potentially pathogenic and are not included in the Qualified Presumption of Safety (QPS) list as the European Food Safety Authority (EFSA) do not recommend the inclusion of Enterococci species in this list [64]. The other four (Bifidobacterium animalis sub. Lactis, Bifidobacterium breve, Bifidobacterium infantis and Saccharomyces boulardii) have been tested alone. B. animalis sub. lactis, has been only used in healthy subjects and shown to modify the gut microbiota composition but only when combined with a prebiotic [24]. Treatment with the other three probiotics has successfully decreased mucositis related symptoms either in cancer patients or in other pathologies. B. breve has decreased adverse effects of chemotherapy treatment, such as fever, diarrhea, and antibiotics consumption in patients with hematologic tumors [32]; B. infantis, has decreased serum levels of proinflammatory cytokines in patients with ulcerative colitis, psoriasis, or chronic fatigue syndrome [28]; and S. boulardii, has decreased colonic mucosa expression of proinflammatory cytokines and decreased postoperative infections [31].

There are possible limitations on the revised essays and the results have to be considered cautiously. For instance, the follow-up period, was not long enough in some cases, between five days and eight weeks [20,21,22,23,25,26,27,28,31,32,34], insufficient time to assess the results [65]. In the studies of Gao et al. [21], and Worthley et al. [24], the number of patients was less than 30, so the results obtained could lack the expected scientific rigor.

## 5. Conclusions

However, despite the limitations, the results of the studies included in this review show that probiotics have been used in different types of cancer patients and they have had a plethora of positive effects including a decrease of the inflammation, the modulation of the immune system, an improved composition of the gut microbiota, with more beneficial bacterial species and less pathogenic ones, and an improvement of the intestinal barrier function. Importantly, they have practically produced no adverse effects.

Thus, probiotics seem to be perfect candidates to ameliorate mucositis in cancer patients, but in order to improve their effectiveness, more clinical trials are needed. It is important to define which probiotic or combination of probiotics is the best one for each type of cancer therapy, discard possible interferences with other components of the treatment and to determine, in each case, which is the best posology.

Taking into account these results, we suggest that a combination of *Biffidobacterium longum*, *Lactobacillus acidophilus*, *Biffidobacterium breve*, *Biffidobacterium infantis,* and *Saccharomyces boulardii* could be a good combination of probiotics to reduce incident rates of mucositis or ameliorate its symptoms in chemo or radiotherapy treated patients, and would be worth being tested in a clinical trial.

We made this suggestion cautiously considering that a lot of data exist about probiotics and mucositis and that, only based on these data, no conclusion can be drawn regarding the superiority of any one particular organism or combination of organisms. Therefore, in this field, there is a good opportunity and a need to develop further investigations

## Figures and Tables

**Table 1:** Mucositis degrees according to WHO [8].

Grade 0	Grade 1	Grade 2	Grade 3	Grade 4
No signs or symptoms	Painless ulcers, edema or mild pain	Painful erythema, edema or ulcers but the patient can eat	Idem grade 2 but unable to eat	Parenteral or enteral feeding is needed

**Table 2:** Summary of reviewed studies on the relationship probiotics and mucositis in cancer patients.

Author, Year	Design	Country	Patients	Pathology	Monitoring	Intervention	Results
Jiang 2008 [20]	Randomized, double-blind, placebo-controlled trial monocentric	China	99 M/F = 58/35 Median age = 51 years	Advanced NC and undergoing CCRT	seven weeks	CCRT and *B.longum*, *L.lactis*, *E.faecium*. (six capsules/day) OM incidence, the short-term curative effect, the immune index, and fecal flora changes.	Probiotic combination reduced the incidence of grade 3 OM and had significantly enhanced the immunity of patients and was beneficial for restoring microbial diversity after the end of CCRT.
Gao 2015 [21]	Randomized prospective placebo-controlled trial.	China	22 M/F = 12/10 Median age = 70 years	CRC	five days perioperative surgery	*B.longum L.acidophyllus*, *Efaecalis* (1:1:1). 6 × 10^7^ CFU/day. Oral probiotics were able to alter the microbial composition and improve gut microbiota in patients with CRC.	Probiotic supplements can effectively alter the composition, richness, and diversity of the gut microbiota
Liu 2010 [22]	Randomized, double-blind, placebo-controlled, prospective trial	China	100 M/F = 59/41 Median age = 65 years	CRC	16 days (six days pre-and 10 days post-operative)	*L.acidophillus, L.plantarum, B.longum* (1 × 10^14^ CFU/day). Preoperative administration could prevent post-operative alterations in intestinal permeability, integrity and microbiota.	Probiotics can improve the integrity of the gut mucosal barrier and balance of the gut microbiota, and they play a role in decreasing the infectious rate.
Gianotti 2010 [23]	Prospective, randomized, double-blind, clinical trial	Italia	31 M/F = 22/11 Median age = 64 years	CRC	six days (three pre- and three post-operative)	*B.longum* and *L.johnsonii* (2 × 10^7^ and 2 × 10^9^ CFU/day). Assess adhesion to the colonic mucosa, reduce concentration of pathogens in stools, and modulate local immune function.	*L.johnsonii* but not *B.longum,* adhere to the colonic mucosa, affect intestinal microbiota by reducing the concentration of pathogens, and modulate local immunity.
Worthley 2009 [24]	Randomized, double-blind, placebo-controlled crossover trial	Australian	20 M/F = 13/7 Median age = 65.5 years	Healthy	16 weeks	(*B.animalis sub.lactis* 5 × 10^9^ CFU/day + 25 g/day HAMS), Synbiotic. Characterized the luminal and biological consequences of these supplements and placed them in the context of colorectal carcinogenesis.	Synbiotic generated a significantly different fecal bacteria profile when compared with either HAMS or *B.animalis sub.lactis* supplementation alone.
Friederich 2011 [25]	Randomized pilot study	Netherlands	31 M/F = 20/11 Median age = 37 years	FAP and IPAA	six weeks	Sulindac (300 mg/day), VSL#3 (9 × 10^11^ CFU/day) + Inulin (12g/day). Endpoints: Risk parameters cell proliferation and GST detoxification capacity in the pouch mucosa. SCFA contents, pH, and cytotoxicity of fecal water.	Study revealed non-significant decreased cell proliferation and increased detoxification capacity after treatment with sulindac or VSL#3/inulin (prebiotic).
Lacouture 2016 [26]	Multicenter, double cohort placebo-controlled randomized phase II trial	USA and Republic. of Korea	173 M/F = 98/75 Median age = 66 years	Advanced NSCLC	four to eight weeks according to treatment	Dacomitinib all cohorts Cohort I: Doxycycline + placebo. Cohort II: AD+ VSL#3. Cohorts I and II assessment incidence of all grade and grade ≥ 2 SDAEI and QoL. Cohort II assessment incidence of all grade and grade ≥ 2 diarrhea and mucositis	Doxycycline was effective as a prophylactic treatment for dacomitinib-induced grade ≥ 2 SDAEI. Both doxycycline and AD reduced the negative impact in patient-reported dermatologic AEs. The probiotic was not effective for preventing diarrhea or mucositis
Hegazy 2010 [27]	Multicenter, placebo-controlled randomized	Egypt	40 M/F = 29/11 Median age = 47 years	UC	eight weeks	*L.delbruekii* and *L.fermentum* 1 × 10^10^ CFU/day. Effect in patients with ulcerative colitis (UC), and their effect on inflammatory mediators and NF-κB activation	Oral supplementation with probiotics could be helpful in maintaining remission and preventing relapse of UC
Groeger 2013 [28]	Randomized, double-blind, placebo-controlled	Ireland	118 M and F = 48 (UC and Psoriasis) F = 83 (CFS and healthy)	UC. Psoriasis. CFS. Healthy.	six - eight weeks	*B. infantis* strain 35,624 1 × 10^10^ CFU/day Assessed the impact on inflammatory biomarker and plasma cytokine levels in UC, CFS, and psoriasis	*B.infantis* strain 35,624, was enough to reduce systemic inflammatory biomarkers in both gastrointestinal and extra-intestinal inflammatory disorders.
Sharma 2012 [29]	Randomized, double-blind, single center, placebo controlled	India	200 M/F = 188/12 Median age = 51 years	HNSCC stage II–IVA	25 months	*L.brevis* strain CD2 2 × 10^9^ efficacy in preventing oral mucositis in patients receiving CRT for HNSCC.	*L.brevis* strain CD2 proved to be safe and efficacious in reducing the incidence of severe oral mucositis in patients with HNSCC undergoing combination radiation and chemotherapy
D Sanctis 2019 [30]	Multicentric, phase III, open label, randomized controlled	Italy	68 M/F = 53/15 Median age = 60 years	HNC	39 months	*L.brevis* strain CD2 2 × 10^9^ primary endpoint was the incidence of grade 3 or 4 oropharyngeal mucositis during radiotherapy treatment	*L.brevis* strain CD2 not able to confirm the beneficial effects in reducing the rate of grade 3–4 RT-induced OM in patients with HNC.
Consoli 2015 [31]	Randomized controlled	Brazil	33 M/F = 15/18 Median age = 55 years	RC	seven days before surgery and was interrupted on the operation day.	*S.boulardii* 0.5 × 10^9^ CFU to assess the role of preoperative treatment on mRNA levels for immunomodulatory cytokines in the colonic mucosa of patients.	*S.boulardii* downregulates both pro- and anti-inflammatory cytokines in the intestinal colonic mucosa with no statistical impact on postoperative infection rates.
Wada 2009 [32]	Randomized, placebo-controlled single-blinded	Japan	40 M/F = 16/24Median age = 6.5 years	IC	one to five weeks	*B.breve* strain Yakult 10^9^ CFU, on its ability to prevent infection, fecal micro flora, and intestinal environments in cancer patients on chemotherapy.	*B.breve strain Yakult* could be an effective approach for achieving clinical benefits in immunocompromised hosts by improving their intestinal environments (mucositis, fever, diarrhea and bacteremia).
Mangell 2012 [33]	Randomized double-blinded, placebo-controlled	Sweden	64 M/F = 36/28Median age = 72 years	RC	six months	*L.plantarum* strain 299v 10^11^ CFU on the intestinal load of potentially pathogenic bacteria, bacterial translocation, and cell proliferation in elective colon surgery.	*L.plantarum* strain 299v was established in the intestine, but no inhibitory effect on enteric bacteria, bacterial translocation, or postoperative complications was found
Ouwehan. 2008 [34]	Randomized double-blinded, placebo-controlled	Finland	47 M/F = 12/35Median age = 71 years	Healthy and regular use of NSAID	six weeks	Synbiotic (lactitol + 2 × 10^9^ CFU *L.acidophyllus* strain NCFM) on improve bowel function and immune function.	Synbiotic twice daily was associated with modest improvement in stool frequency without any side-effects and improved microbiota composition and mucosal.

**M/F**: Number males and females. **NC**: Nasopharyngeal Carcinoma. **CCRT**: Concurrent Chemoradiotherapy. **OM**: Oral mucositis. **CRC**: Colorectal cancer. **B**: *Bifidobacterium*. **L**: *Lactobacillus*. **E**: *Enterobacteria*. **CFU:** Colony-forming units. **HMAS (prebiotic):** High-Amylose Maize Starch. **FAP**: Familial adenomatous polyposis. **IPAA**: Ileal pouch anal anastomosis. **Sulindac**: Non-steroidal anti-inflammatory drug. **VSL#3**: Mix probiotic. **SCFA**: Short chain fatty acid. **GST**: Glutathione S-transferase. **NSCLC**: Non-small cell lung cancer. **QoL**: Quality of life. **SDAEI**: Select dermatologic adverse events of interest. **AD**: Alclometasone dipropionate**. Dacomitinib**: Inhibitor of the human epidermal growth factor receptor (HER). **AEs**: Adverse events. **Doxycycline**: Broad-spectrum antibiotic. **UC**: Ulcerative colitis. **CFS:** Chronic fatigue syndrome. **HNSCC**: Head and neck squamous cell carcinoma **CRT**: Chemo-radiotherapy. **HNC**: Head and neck carcinoma. **RT**: Radiation therapy. **NF κB**: Nuclear factor kappa-light-chain-enhancer of activated B cells. **mRNA**: Messenger RNA. **RC**: Resection colon. **IC**: Immunocompromised. **NSAID**: Non-steroidal anti-inflammatory. **S**: Saccharomyces. **Lactitol (prebiotic)**: Disaccharide.

**Table 3:** Assessment of the methodological quality of the studies analyzed by means of the 25 items of the CONSORT 2010.

	1	2	3	4	5	6	7	8	9	10	11	12	13	14	15	16	17	18	19	20	21	22	23	24	25	T	%
Jiang [20]	1	1	1	1	1	1	1	1	1	1	1	0.75	1	1	1	1	1	1	1	0	0.8	1	1	0.5	1	22.5	90
Gao [21]	0	1	1	1	1	1	0	0.5	0	0.5	0.5	1	1	0.5	0.5	1	1	NA	0.5	0	1	1	0	0	1	15	62
Liu [22]	1	1	1	1	1	1	1	1	1	1	1	1	1	0.5	1	1	1	1	1	1	1	1	1	1	1	24.5	98
Gianotti [23]	1	1	0.5	1	1	0.5	0	1	1	1	1	1	1	0	1	1	0.5	NA	0	1	1	1	1	0	1	18.5	77
Worthley [24]	0	0.5	0.5	1	1	0.5	0	0	0	1	0.5	1	0.5	0	0	1	0.5	NA	1	1	1	1	1	1	1	15	62
Lacouture [26]	0.5	1	1	0.5	1	1	0.5	0	0	0	0.5	1	0.75	0	1	1	0.5	1	1	0	1	1	1	0	1	16.2	65
Hegazy [27]	0.5	1	0.5	1	1	0.5	0	0	0	0	0	0.5	0	0	1	1	0.5	1	0	0	1	0.5	0	0	0	9.5	38
Groeger [28]	0.5	1	0.5	1	1	0.5	0	0	0	1	0.5	1	0	0	0	1	0.5	NA	0	0	1	1	0	0	0	10.5	44
Sharma [29]	1	1	1	1	1	1	0.5	1	1	1	1	0.5	1	0.5	1	1	0.5	1	1	0	1	1	1	1	1	22	88
De Sanctis [30]	0.5	1	1	1	1	1	1	1	0	1	NA	1	0.75	1	1	1	0.5	1	1	1	0.5	1	1	0	1	20.2	84
Consoli [31]	1	1	1	1	1	1	1	1	1	1	NA	0.5	1	0	1	1	0.5	NA	1	1	1	1	1	0	1	20	87
Wada [32]	0.5	1	1	1	1	1	0	0.5	0	0	0	1	0.5	1	0.5	1	0.5	1	1	1	0.5	1	0	0	0	14	56
Mangell [33]	0.5	1	1	1	1	0.5	1	0	1	1	0.5	0.5	0.5	0	1	1	0.5	NA	1	1	0.5	1	0	0	1	16.5	69
Ouwehand [34]	0.5	1	1	1	1	0.5	0	0	0	1	0.5	0.5	0.5	0	1	1	1	1	0	0	1	1	0	0	1	14.5	58
Friederich [25]	0.5	1	0.5	1	1	0.5	0	0	0	1	0	0.5	0.5	0.5	1	1	0.5	1	1	1	1	1	0	1	1	16	64
